# TREM2 promotes macrophage polarization from M1 to M2 and suppresses osteoarthritis through the NF-κB/CXCL3 axis

**DOI:** 10.7150/ijbs.91519

**Published:** 2024-03-11

**Authors:** Chao Fang, Rui Zhong, Shuai Lu, Gang Yu, Zhilin Liu, Chengyuan Yan, Jingyu Gao, Yang Tang, Yingming Wang, Qichun Zhao, Xinzhe Feng

**Affiliations:** 1 Department of Orthopedics, The First Affiliated Hospital of USTC, Hefei, 230001, China.; 2 Department of Joint Bone Disease Surgery, Changhai Hospital, Naval Medical University, Shanghai, 200433, China.

**Keywords:** Osteoarthritis, TREM2, Macrophage, NF-κB, CXCL3

## Abstract

**Objective:** Osteoarthritis (OA) is the most prominent chronic arthritic disease, affecting over 3 billion people globally. Synovial macrophages, as immune cells, play an essential role in cartilage damage in OA. Therefore, regulating macrophages is crucial for controlling the pathological changes in OA. Triggering receptor expressed on myeloid cells 2 (TREM2), as expressed on immune cell surfaces, such as macrophages and dendritic cells, has suppressed inflammation and regulated M2 macrophage polarization but demonstrated an unknown role in synovial macrophage polarization in OA. This study aimed to investigate TREM2 expression downregulation in OA mice macrophages. Furthermore, the expression trend of TREM2 was associated with polarization-related molecule expression in macrophages of OA mice.

**Results:** We used TREM2 knockout (TREM2-KO) mice to observe that TREM2 deficiency significantly exacerbated the joint inflammation response in OA mice, thereby accelerating disease progression. Separating macrophages and chondrocytes from TREM2-KO mice and co-cultivating them significantly increased chondrocyte apoptosis and inhibited chondrocyte proliferation. Further, TREM2 deficiency also significantly enhanced phosphatidylinositol 3-kinase(PI3K)/AKT signaling pathway activation, increasing nuclear factor kappa light chain enhancer of activated B cells (NF-κB) signaling and C-X-C Motif Chemokine Ligand 3 (CXCL3) expression. Furthermore, NF-κB signaling pathway inhibition significantly suppressed arthritis inflammation in OA mice, thereby effectively alleviating TREM2 deficiency-related adverse effects on chondrocytes. Notably, knocking down CXCL3 of TREM2-KO mice macrophages significantly inhibits inflammatory response and promotes chondrocyte proliferation. Intravenous recombinant TREM2 protein (soluble TREM2, sTREM2) injection markedly promotes macrophage polarization from M1 to M2 and improves the joint tissue pathology and inflammatory response of OA.

**Conclusion:** Our study reveals that TREM2 promotes macrophage polarization from M1 to M2 during OA by NF-κB/CXCL3 axis regulation, thereby improving the pathological state of OA.

## Introduction

Osteoarthritis (OA) is the most prominent joint disorder characterized by joint cartilage degeneration, which affects millions of people globally^1^. Over time, OA occurs as the protective cartilage that cushions the ends of bones wears away, often affecting the knee, hip, and distal finger joints^2^. The occurrence of OA is mainly related to aging, obesity, excessive joint activity (such as frequent and intense joint movements), joint injuries, genetic factors, high pressure within the bones, osteoporosis, metabolic and endocrine abnormalities, and so on^2^, ^3^. OA is expected to become the primary cause of disability in the general population by 2030, and approximately 35% of individuals are predicted to eventually develop this condition^4^. In the early stage of OA, the increase in cartilage water content leads to cartilage degradation, causing matrix swelling and intensifying inflammatory reactions. This gradually leads to cartilage calcification and thinning of the joint surface. When OA progresses to its late stage, other treatments are no longer effective, and total joint replacement surgery becomes the only option^5^. This will result in significant medical expenses and severely affect the patient's quality of life, and rarely achieve full joint functionality. Therefore, intervening in the early stages of OA can reduce its impact on individuals and the socioeconomic level. Recent research has shown that, besides mechanical load, inflammation (especially synovitis) also has a significant impact on the progression of OA, particularly noteworthy are its various effects on early-stage OA^6^.

Macrophages play a crucial role in innate immunity, and their polarized phenotype is associated with OA progression^7^. Macrophages are categorized into classically activated M1 macrophages and alternatively activated M2 macrophages, which produce different stimuli responses from the microenvironment^8^. M1 macrophages secrete large amounts of proinflammatory cytokines and mediators, such as tumor necrosis factor α (TNF-α), interleukin (IL)-1, IL-6, IL-12, and cyclooxygenase-2 (Cox-2), after activation with interferon-γ (IFN-γ) and lipopolysaccharide (LPS) or TNF-α^8^, ^9^.

Cartilage fragments, aggregated proteins, fibrous connective proteins, and intracellular proteins of necrotic cells in OA serve as danger-associated molecular patterns, which stimulate macrophage activation and aggregation, thereby enhancing their ability to synthesize inflammatory cytokines and metalloproteinases^10^. Wu et al. revealed that depleting both M1 and M2 macrophages does not relieve OA severity, but rather worsens the inflammatory response^11^. The research conducted by Zhang et al. found that polarization of M1 macrophages exacerbates cartilage degradation and osteophyte formation in OA mice. Conversely, polarization of M2 macrophages weakens the related symptoms in OA mice^12^. This indicated that macrophage polarization was closely associated with the occurrence and progression of OA.

The triggering receptor expressed on myeloid cell (TREM) family is a group of cell surface receptors that mediate the regulation of inflammatory responses, which attracted widespread attention from researchers in recent years^13^. The TREM family includes TREM1, TREM2, and TREM3^13^. TREM2, as a cell surface receptor of the immunoglobulin superfamily, has promoted M2 polarization of macrophages and participated in regulating inflammation by reducing macrophage infiltration^14^-^18^. TREM2 plays an important role in macrophage polarization and regulation of inflammatory response, and there is extensive research on its involvement in diseases such as Alzheimer's disease^19^, ^20^, metabolic syndrome^21^, and cancer^22^, ^23^. In recent years, research on the role of macrophage TREM2 in arthritis has also been gradually conducted. Alivernini et al.'s study demonstrated that in rheumatoid arthritis (RA), macrophages with high expression of TREM2 were effective producers of anti-inflammatory lipid mediators and induced reparative responses in synovial fibroblasts in vitro^24^. Sebastian et al.'s study indicated that in in injured knee joints with OA, TREM2^+^ macrophages were recruited to the site of tissue damage, and macrophages expressing high levels of TREM2 may be involved in the phagocytosis and subsequent tissue repair after injury^25^. The high expression of TREM2 in macrophages has significant implications for the recovery and treatment of arthritis, thus making it extremely urgent to conduct in-depth research on this process.

This study used destabilization of medial meniscus (DMM) surgery to develop the OA mouse model and assessed the changes in macrophage TREM2 expression. We evaluated the polarization of macrophages in OA by detecting the iNOS (M1 macrophage marker) and CD206 (M2 macrophage marker) levels. Additionally, we used TREM2 knockout (TREM2-KO) mice on the C57BL/6 background to further investigate the mechanism by which TREM2 regulates macrophage polarization and inflammatory response in OA. We revealed a significantly decreased TREM2 expression in OA mice, while M1 macrophages significantly increased. TREM2-KO mice demonstrated a significant increase in M1 macrophages, aggravated cartilage degradation, and enhanced inflammatory response. Moreover, TREM2-KO mice macrophages significantly enhanced chondrocyte apoptosis and inhibited their proliferation. On the contrary, supplementing recombinant TREM2 to C57BL/6 wild-type (WT) mice significantly induced M2 polarization of synovial macrophages, suppressed inflammation, and alleviated OA. These results indicate TREM2 as a new therapeutic target for OA treatment.

## Materials and Methods

### Animal models

All female WT mice and TREM2-KO mice were purchased from Shulaibao Biotech (Wuhan, China). We placed 10 ± 2 weeks old C57BL/6 mice weighing 18 - 22 g in a specific pathogen-free environment, with a temperature of 23°C ± 2°C, a 12-h light-dark cycle, and unrestricted access to food and water. We used 1% pentobarbital sodium (50 mg/kg, IP) as an anesthetic to minimize pain throughout all procedures. We used the method of surgically inducing stable DMM to develop an OA mouse model 12. These mice have at least one week to adapt before initiating the experiment. Afterward, these mice were randomly categorized into groups to study the effects of TREM2 on OA. In the subsequent TREM2 treatment experiments, the effects of TREM2 on OA mice were investigated by intravenous injection of soluble TREM2 (sTREM2) in the mouse tail.

### Cell culture

Primary macrophages and chondrocytes were extracted from the knee joints of mice by removing the articular cartilage and placing it in a digestion solution (which consisted of 1% collagenase II [Sigma], 0.5% trypsin-EDTA, and 1% penicillin/streptomycin in Dulbecco's Modified Eagle Medium (DMEM) and allowing it to incubate for 2 h. After filtering the digested suspension using a Corning of 40 μm cell filter (Sigma), it is centrifuged at 1500 rotations per minute (rpm). Afterward, the resulting cell pellet is resuspended in DMEM and supplemented with 10% fetal bovine serum (BI) and 1% penicillin/streptomycin. The cells were then seeded onto a 6-well plate with a density of 3 × 10^6^ cells per well, and then kept at a temperature of 37°C in a constant temperature incubator with 5% CO_2_.

### Cell stimulation and co-cultivation

Co-culture the separated mouse synovial macrophages with articular chondrocytes to investigate the effects of TREM2 on macrophage polarization and chondrocyte function in OA. It is known that LPS and IFN-γ induce macrophage differentiation into M1 type, triggering the secretion of pro-inflammatory factors and inducing an inflammatory reaction, which is similar to the immune process in OA. Therefore, we firstly cultured isolated WT and TREM2-KO mouse macrophages in a medium containing LPS (200 ng/mL) and IFN-γ (2.5 ng/mL) for 12 h, and then co-cultured them with chondrocytes for 24 h. BAY 11-7082 (HY-13453, MedChemExpress, USA) is an NF-κB inhibitor, which we used to study the mechanism of TREM2 on macrophage polarization and its effects on OA. Simply put, when LPS and IFN-γ-induced TREM2-KO macrophages were co-cultured with chondrocytes, the addition of BAY 11-7082 (100 μM) was performed, and after 24h, chondrocyte function was assessed.

### Hematoxylin and eosin (H&E), Safranin O/Fast Green (SOFG), and immunohistochemistry staining

The newly dissected mouse knee joint was fixed in 4% formaldehyde, refrigerated at 4°C for 24 h, and decalcified with 14% ethylenediaminetetraacetic acid (EDTA) (pH 7.4) at 25°C for 30 days. The organization is embedded in paraffin, sliced continuously (with a 3-micrometer thickness), and stained with either H&E or SOFG. Ultimately, the images are captured using an optical microscope. The following steps are performed to stain different proteins: dewaxing, rehydration, antigen detection, and blocking with 5% bovine serum albumin (BSA) for 1 h at room temperature (RT: 26°C). Afterward, the primary antibody is incubated overnight at 4°C, and then the secondary antibody labeled with biotin is incubated at RT for 1 h. Finally, the microscope is used to record the images.

### Western blot assay

Total proteins from the lung tissues or cells of mice were extracted using the RIPA lysis buffer (Solarbio, China). The BCA protein concentration determination kit (Beyotime, China) was used to determine protein concentration. The proteins from different samples were separated by SDS-PAGE and transferred to a polyvinylidene fluoride membrane. The membrane was then sealed with 5% BSA at RT for 2 h. Subsequently, it was incubated overnight at 4°C with different primary antibodies (1:1,000-2,000). After removing unbound primary antibodies from the membrane, the corresponding HRP-conjugated Abs (1:5,000-8,000) were incubated at RT for 1.5 h. Finally, the ECL detection kit (Beyotime, China) and the image processing software Image J were used to analyze the film for grayscale values of the bands.

### Flow cytometry

The Annexin V-FITC/ Propidium Iodide (PI) Apoptosis Detection Kit (Vazyme, China) was used to detect the cell apoptotic process 26, 27. Approximately 1 × 10^6^ cells were taken, washed with PBS, and slowly treated with 3 ml of 70% ethanol for overnight fixation at 4°C. Afterward, the cells were gently mixed with a combination of Annexin V-FITC and PI, stirred, and left at RT for 15 min. The FACSCalibur flow cytometer (BD Biosciences, USA) was used to examine the distribution of cells, and finally, the FlowJo software was utilized for analysis.

### Terminal deoxynucleotidyl transferase-mediated dUTP nick end labeling (TUNEL) assay

The TUNEL assay kit (Beyotime, China) was used for experiments to analyze cellular apoptosis, following the manufacturer's instructions. Subsequently, a fluorescence microscope was used to capture images of the samples for further analysis.

### Micro-computed tomography (Micro-CT)

The Micro-CT technology was applied to scan and analyze the bone tissues of mice. After constructing the mouse model, determined the scanning range from the distal end to the middle of the femur, with a total of 400 layers and a thickness of 9 μm per layer. The scanning parameters are set at 46 kV voltage, 75 μA current, and a scanning time of 10 minutes. Finally, Nrecon software was used to construct a three-dimensional (3D) visualization image.

### GO and KEGG analysis

Differential genes were uploaded to the DAVID database (https://david.ncifcrf.gov/summary.jsp) 28 in order to conduct GO and KEGG analysis. Adjust the P-values using the Benjamini Hochberg method.

### Statistical analysis

GraphPad Prism 9.0 software was used for all analyses. The mean ± standard error of the mean (n ≥ 3) was used to present the experiments. A nonparametric *t*-test was conducted to compare the two groups. One-way analysis of variance and Turkey's test were used to assess multiple groups and determine statistical differences. Statistical significance was defined as a *p*-value of <0.05.

## Results

### TREM2 expression decreases in OA mice

**Figure 1:**
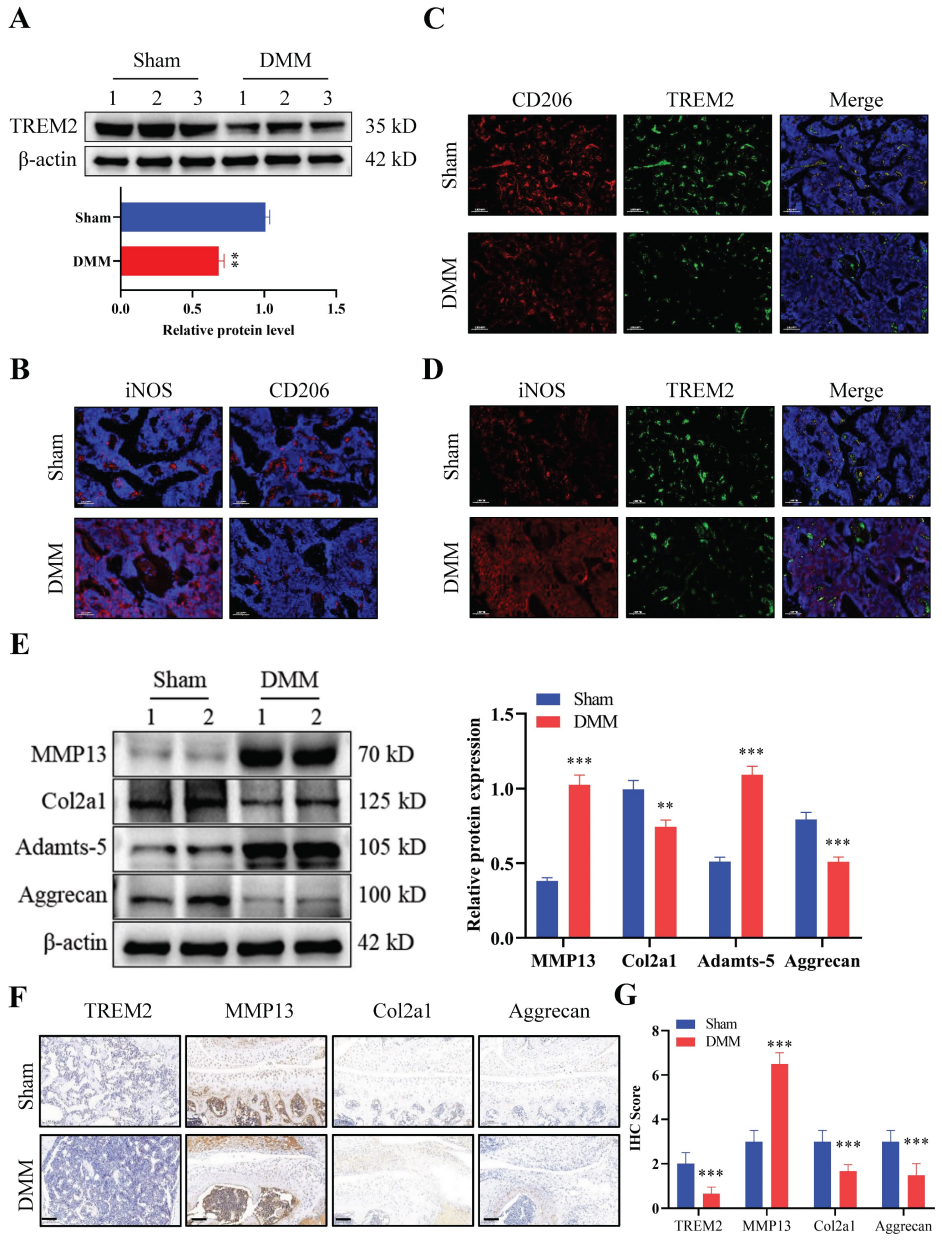
** Triggering receptor expressed on myeloid cell 2 (TREM2) expression increases in osteoarthritis (OA) mice.** A, Western blotting was used to detect TREM2 expression levels in WT and OA model (destabilization of medial meniscus [DMM] group) mice tissues. The endogenous control used was β-actin. 1, 2, and 3 represent the different samples. The quantitative display of relative TREM2 expression levels in samples related to β-actin. **P < 0.01, compared to the Sham group. B, Sham and DMM mouse synovial iNOS and CD206 representative immunofluorescence images. Scale: 100 μm. C, CD206 and TREM2 representative immunofluorescent images in the synovium of mice from the Sham and DMM groups, as well as co-localization immunofluorescent images. Scale: 100 μm. D, Representative immunofluorescence images of iNOS and TREM2 in the synovium of mice in the Sham and DMM groups, as well as co-localization immunofluorescence images. Scale: 100 μm. E, The protein expression levels involved in cartilage matrix degradation (MMP13 and Adamts-5) and remodeling (Col2a1 and Aggrecan) were detected, using Western blotting, in the tissues of Sham and DMM mice. The endogenous control used was β-actin. 1 and 2 represent the different samples. The right figure quantitatively shows the relative expression levels of different proteins related to β-actin in the samples. **P < 0.01, ***P < 0.001, compared to the Sham group. F, Immunochemical images of TREM2, MMP13, Col2a1, and Aggrecan in Sham and DMM mouse tissues. Scale: 100 μm. G, Relative quantitative results of immunohistochemistry. ***P < 0.001, compared to the Sham group.

We first developed an OA mouse model (DMM group) to explore the role of TREM2 in OA development. The results of tissue staining indicated high levels of synovial hyperplasia and abundant cell infiltration in the synovial tissue of OA mice (Fig. S1A-B), with lower International Cartilage Repair Society (ICRS) scores compared to the Sham group (Fig. S1C). Firstly, we tested the expression levels of TREM2 in two groups and revealed a significant decrease in TREM2 expression in the DMM group (Fig. 1A). We further identified the accumulation and phenotypic characteristics of macrophages in the OA synovial tissue. The DMM group demonstrated a significant increase in the number of cells positive for iNOS (a marker of M1 macrophages), while the number of cells positive for CD206 (a marker of M2 macrophages) did not significantly change compared to the Sham group (Fig. 1B). Compared with the Sham group, the expression of CD206 and TREM2 expression were significantly reduced in DMM group (Fig. 1C). In contrast, the level of iNOS was increased in the DMM group inconsistently changed with TREM2 (Fig. 1D). These results indicate that M1, instead of M2 polarized macrophages, accumulate in the synovium. Matrix metalloproteinase (MMP)13 and Adamts-5 are the two most important enzymes that contribute to cartilage degradation. The expression levels of these two proteins are significantly increased in the DMM group, while that of Col2a1, Col1a1, and Aggrecan, which are responsible for stabilizing cartilage morphology and maintaining cartilage differentiation, are significantly reduced (Fig. 1E, S1D). Concurrently, the immunohistochemistry experiment obtained similar results. Notably, TREM2 was demonstrated in the immunohistochemical results, with a significantly lower expression in the DMM group than in the Sham group (Fig. 1F-G). In summary, these findings indicate that TREM2 expression is closely related to the function of OA synovial macrophages and is likely to have a potential role in OA development.

### TREM2 deficiency exacerbates inflammatory response and suppresses macrophage M2 polarization

**Figure 2:**
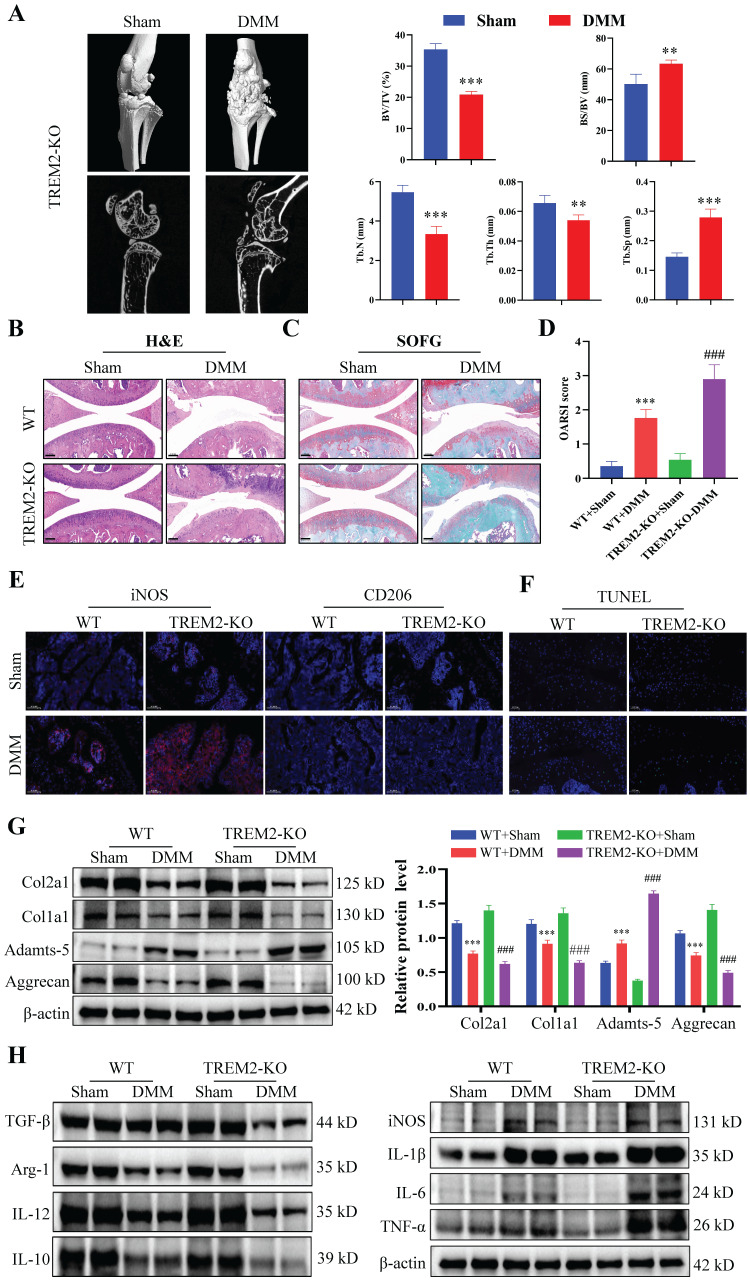
** TREM2 deficiency exacerbates inflammatory response and suppresses macrophage M2 polarization.** A, The representative images of bone microstructure in the Sham and OA models in WT and TREM2-KO mice were obtained using Micro-CT. The right-hand side shows the statistical charts of the testing results for bone tissue and trabecular bone, including bone tissue volume to tissue volume ratio (BV/TV), bone surface area to bone volume ratio (BS/BV), trabecular number (Tb-N), trabecular thickness (Tb-Th), and trabecular separation (Tb-Sp). **P < 0.01, ***P < 0.001, compared to the Sham group. B-C, Representative images of Safranin O/Fast Green (SOFG) stained cartilage and H&E staining synovium from the four different groups of mice, including wild-type (WT) + Sham group, WT + DMM group, TREM2-KO + Sham group, and TREM2-KO + DMM group. Scale: 100 μm. D, Quantification of the scoring for synovitis and OARSI (n = 5) in the four groups. ***P < 0.001, compared to the WT + Sham group. ^###^*P* < 0.001, compared to the TREM2-KO + Sham group. E, The representative immunofluorescence images of iNOS and CD206 positive cells in the synovium of the four groups of mice. Scale: 100 μm. F, The Terminal deoxynucleotidyl transferase-mediated dUTP nick end labeling (TUNEL) detection results of apoptotic cells in the synovial membrane of the four groups of mice. Scale: 100 μm. G, Western blotting detected the expression levels of the related proteins involved in extracellular matrix maintenance and degradation of the synovial tissue in the four groups of mice. ***P < 0.001 compared to the WT + Sham group, ^###^*P* < 0.001 compared to the TREM2-KO + Sham group. H, Western blotting was used to detect the expression levels of inflammatory cytokines and macrophage polarization markers (iNOS and Arg-1) in the synovial tissue of the four groups of mice.

We further investigated the functional role of TREM2 in OA development. The TREM2-KO mice (TREM2-KO group) were used for the subsequent experiments. Micro-CT and statistical results on bone tissue revealed that the DMM group demonstrated a significant decrease in bone mass compared to the Sham group, manifested by a reduced bone tissue volume to tissue volume ratio (BV/TV) and increased bone surface area to bone volume ratio (BS/BV). Concurrently, the trabeculae number (Tb-N) decreased, the trabeculae thickness (Tb-Th) decreased, and the trabecular separation (Tb-Sp) increased (Fig. 2A). Consistent with previous results, the synovial tissues of both WT and TREM2-KO mice demonstrated high synovial hyperplasia levels and abundant cellular infiltration after developing a mouse model of OA through DMM (Fig. 2B--C). Moreover, the absence of TREM2 considerably exacerbated this trend and resulted in a higher synovial inflammation score (Fig. 2D).

The immunofluorescence results revealed that the iNOS expression was the highest, while that of CD206 was the lowest in DMM mice that lack TREM2 (Fig. 2E). This indicates that the absence of TREM2 can significantly promote M1 macrophages polarization while inhibiting M2 macrophages. Further research results revealed that the absence of TREM2 significantly aggravated cartilage damage in OA. This was manifested by the absence of TREM2 which significantly enhanced the apoptosis of chondrocytes in the tissue (Fig. 2F). Additionally, the expression of MMP13, ColX, and Adamts-5 is significantly increased in the TREM2-KO-DMM group compared with the WT-DMM group, while Col2a1, Col1a1, and Aggrecan expressions were significantly decreased, thereby further damaging the function of the cartilage (Fig. 2G, S2A). We detected the expression levels of inflammatory factors in the synovial tissues of each group of OA mice to evaluate whether TREM2 affects the inflammation response in OA. IL-1β, IL-6, and TNF-α, as typical proinflammatory cytokines, were tested. The results revealed that the levels of these three factors were significantly increased in the TREM2-KO-DMM group compared to the WT-DMM group (Fig. 2H). TGF-β and IL-10 are powerful anti-inflammatory cytokines, and their expression was significantly decreased in the TREM2-KO-DMM group (Fig. 2H). Interestingly, despite IL-12 being a pro-inflammatory factor, the absence of TREM2 did affect its expression. Additionally, the results of western blot further demonstrated that the loss of TREM2 promoted M1 macrophage polarization and inhibited M2 macrophage polarization, which was manifested as increased iNOS expression in the TREM2-KO-DMM group and decreased Arg-1 expression (M2 macrophage marker molecule) (Fig. 2H). Moreover, the results of immunohistochemical experiments indicated that the absence of TREM2 significantly enhanced MMP13 expression and decreased Aggrecan expression (Fig. S2B-C).

### The absence of TREM2 exacerbates chondrocyte apoptosis and inhibits their proliferation, thereby disrupting the normal functions of chondrocytes

**Figure 3:**
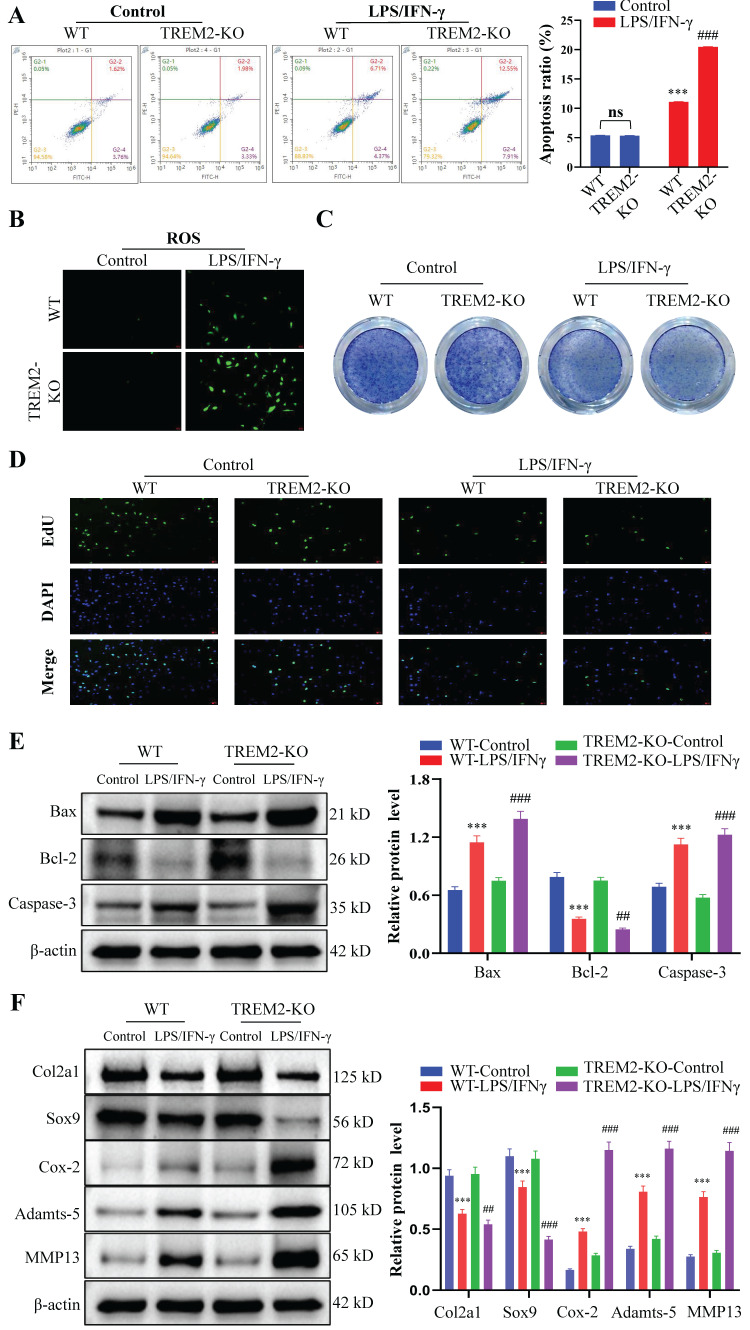
** The absence of TREM2 exacerbates chondrocyte apoptosis and inhibits their proliferation, thereby disrupting normal chondrocyte functions.** A, Bone marrow macrophages were separated from WT and TREM2-KO mice and co-cultured with chondrocytes, with or without LPS/IFN-γ induction. Flow cytometry is used to detect chondrocyte apoptosis. The right image shows the quantitative and statistical results of the apoptosis ratio of each group of chondrocytes. ***P < 0.001, compared to the WT-Control group, ^###^*P* < 0.001, compared to the TREM2-KO-Control group. B, Reactive oxygen species (ROS) detection in the co-culture medium of macrophages and chondrocytes of each group. C, Toluidine blue (TB) staining was performed on various groups of chondrocytes. D, EdU was used to assess the cell proliferation of each group. E, Western blotting is used for protein detection related to chondrocyte apoptosis in different groups. ***P < 0.001, compared to the WT-Control group, ^##^*P* < 0.01, ^###^*P* < 0.001, compared to the TREM2-KO-Control. F, Western blotting was used to detect the protein levels associated with cartilage extracellular matrix formation and degradation in each group. ***P < 0.001, compared to the WT-Control group, ^##^*P* < 0.01, ^###^*P* < 0.001, compared to the TREM2-KO-Control group.

The proliferation, apoptosis, and phenotypic changes (such as cell hypertrophy and matrix calcification) of chondrocytes play a crucial role in OA occurrence and development process^29^, ^30^. Therefore, we then focused on chondrocyte regulation by macrophage TREM2. We obtained WT and TREM2-KO mice bone marrow macrophages through fractionation and cultured them in a medium that contains LPS (200 ng/mL) and IFN-γ (2.5 ng/mL) for 12 h. Afterward, they were co-cultured with chondrocytes for 24 h. Generally, LPS and IFN-γ induce macrophage differentiation into M1 type^31^. The results revealed that macrophages significantly promote apoptosis in chondrocytes when co-cultured after induction with LPS and IFN-γ (Fig. 3A). Additionally, the release of reactive oxygen species (ROS) increased in the co-culture environment (Fig. 3B). Toluidine blue staining revealed a significant reduction in the number of chondrocytes (Fig. 3C). Concurrently, the proliferative ability of chondrocytes was significantly inhibited (Fig. 3D). Notably, LPS and IFN-γ-induced TREM2-KO macrophages cause more severe damage to chondrocytes than normal, as evidenced by more severe chondrocyte apoptosis, increased ROS release, decreased chondrocyte population, and pronounced inhibitory effects on chondrocyte proliferation (Fig. 3A-D). The above results indicate that the absence of TREM2 significantly enhances macrophage polarization to the M1 type, thereby exacerbating the damage to chondrocyte function. Simultaneously, the experimental results of the Western blot have similar persuasiveness. This is manifested as the significant inhibition of anti-apoptotic molecule expression (such as Bcl-2) and promotion of proapoptotic molecule (such as Bax and Caspase-3) expression in the absence of TREM2 under LPS and IFN-γ induction (Fig. 3E). Additionally, the macrophages with TREM2 deficiency demonstrated significantly enhanced expression of molecular markers (such as Cox-2, Adamts-5, and MMP13) that promote chondrocyte destruction and decreased molecular marker expression (such as Col2a1 and Sox9) that maintain chondrocyte function after LPS and IFN-γ induction (Fig. 3F).

### TREM2 regulates the PI3K/Akt/NF-κB signaling pathway and affects CXCL3 expression

**Figure 4:**
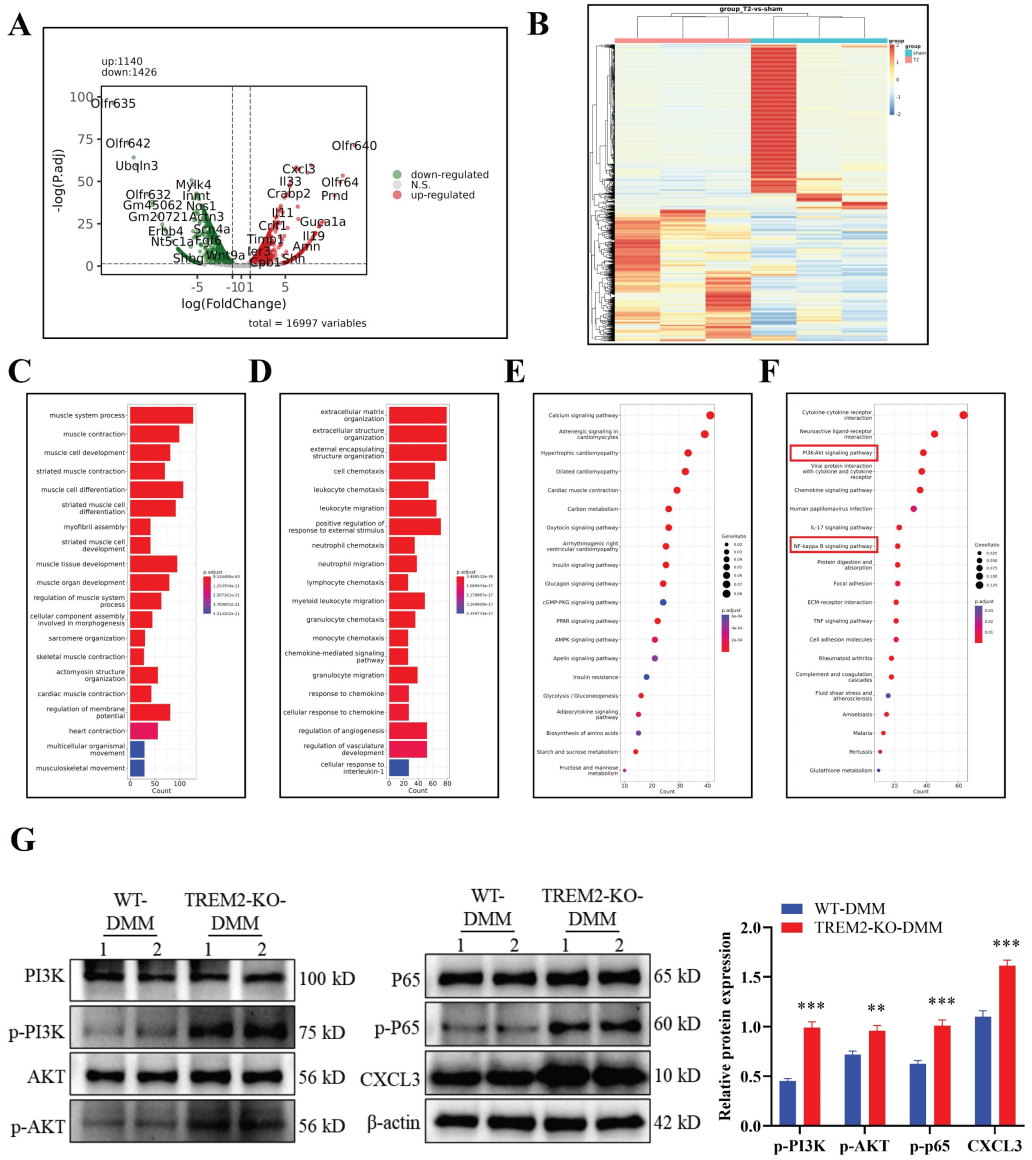
** TREM2 regulates the PI3K/Akt/NF-κB signaling pathway and affects CXCL3 expression.** A, The differential gene analysis schematic derived from transcriptome sequencing of tissue samples obtained from developing the DMM model in WT and TREM2-KO mice. B, Heatmap. C-D, Gene Ontology enrichment analysis of the biological functions of differentially expressed genes (DEGs). E-F, Genes and Genomes Encyclopedia (KEGG) analyzes the biological functions of DEGs. The red highlighted area is the PI3K/AKT and NF-κB signaling pathways identified and paid attention to. G, Using Western blotting detects PI3K/AKT and NF-κB (p65) signaling pathway activation levels. The endogenous control used was β-actin. **P < 0.01, ***P < 0.001, compared to the WT-DMM group.

After developing the OA model in WT and TREM2-KO mice, we directly obtained tissues for transcriptomic sequencing to identify differentially expressed genes and perform GO and KEGG pathway analyses (Fig. 4A--F).

The result revealed that C-X-C Motif Chemokine Ligand (CXCL3) was significantly upregulated in TREM2-KO-DMM mice (Fig. 4A). CXCL3 is a type of cell chemotactic factor, playing an important role in cell chemotaxis in inflammation and immune response^32^. The changes in genes related to OA inflammation and fibrosis were analyzed according to the sequencing results, as well as the signaling pathways that regulate OA. The results indicated significant differences in the phosphatidylinositol 3-kinase (PI3K)/AKT and NF-κB signaling pathways (Fig. 4F). A study has revealed that NF-κB activation is involved in iNOS expression induction by LPS and M1-type macrophage polarization^33^. The PI3K/Akt signaling pathway regulates various processes and is involved in the cellular and extracellular matrix changes in OA pathogenesis^34^. Furthermore, the membrane protein PI3K directly or indirectly induces AKT phosphorylation under stimulation from cytokine receptors and other factors, thereby activating NF-κB and increasing MMP production in chondrocytes^35^. The western blot experiment results further indicate that the absence of TREM2 significantly enhances PI3K/AKT/NF-κB (P65) signaling pathway activation in OA mice (Fig. 4G). Concurrently, CXCL3 expression is significantly increased (Fig. 4G).

### TREM2 regulates chondrocyte function through the NF-κB/CXCL3 axis

**Figure 5:**
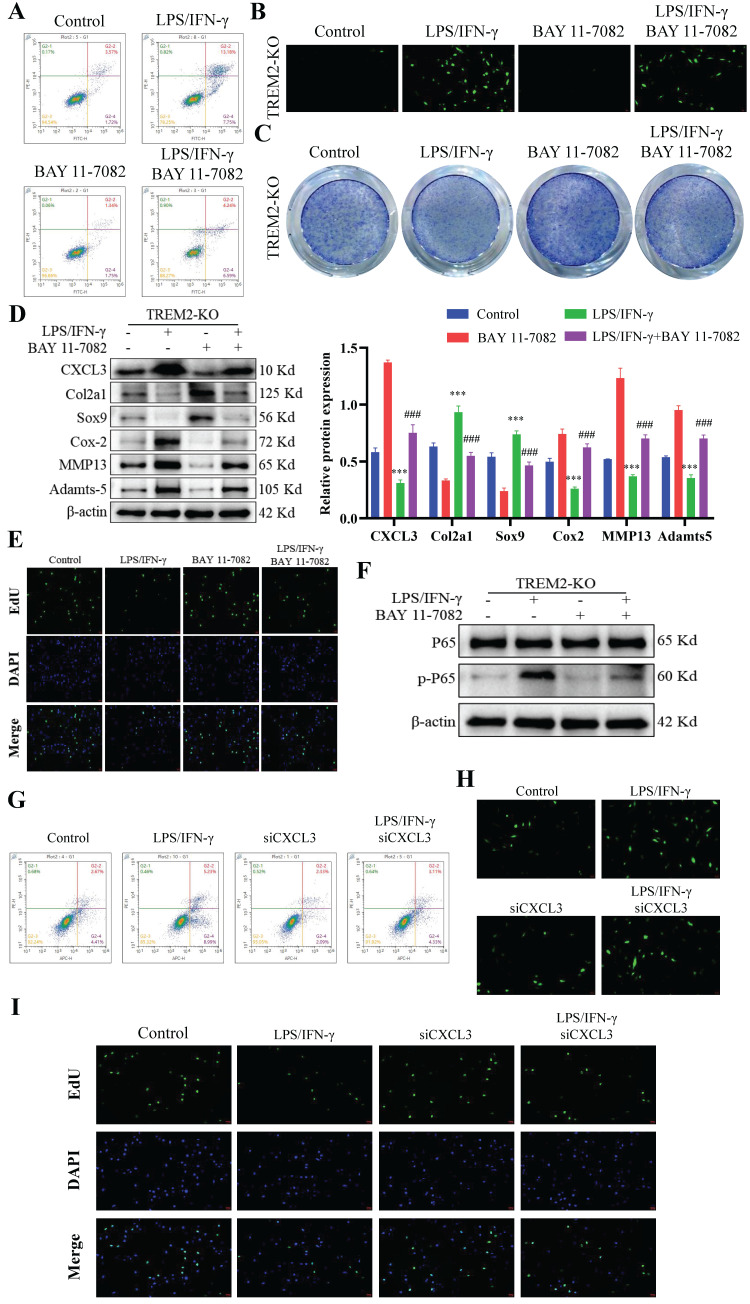
** TREM2 regulates chondrocyte function through the NF-κB/CXCL3 axis.** A, BAY 11-7082 is used to inhibit NF-κB signaling pathway activation. TREM2-KO bone marrow macrophages were isolated and pretreated with LPS/IFN-γ and/or BAY 11-7082 before co-culturing them with chondrocytes. Flow cytometry was used to detect chondrocyte apoptosis. B, Detection of the ROS content in the co-culture medium of macrophages and chondrocytes in each group. C, TB staining was performed on various groups of chondrocytes. D, The protein content of CXCL3 in each group and proteins associated with maintenance and degradation of cartilage extracellular matrix were measured. The statistical graph on the right depicts the relative protein content. ***P < 0.001, compared to the control group, ^###^*P* < 0.001, compared to the BAY 11-7082 group. E, Cell proliferation of each group was assessed by EdU. F, Western blotting is used to detect NF-κB (p65) phosphorylation levels in different groups of macrophages. G, The transfection of small interfering RNA (si-CXCL3) was used to decrease CXCL3 expression in TREM2-KO macrophages. LPS/IFN-γ pretreated normal or low CXCL3-expressing TREM2-KO macrophages were used and co-cultivated with chondrocytes. Flow cytometry was used to detect chondrocyte apoptosis in each group. H, Detection of the ROS content in the co-culture medium of macrophages and chondrocytes in each group. I, Cell proliferation of each group was assessed by EdU.

BAY 11-7082 is an inhibitor that hinders IκBα phosphorylation and NF-κB and is commonly used to investigate the function of the NF-κB signaling pathway^36^-^38^. Our study revealed that LPS and IFN-γ-induced TREM2-KO macrophages, when co-cultured with chondrocytes, demonstrated significant inhibition of their destructive effects on chondrocyte function upon BAY 11-7082 addition. This manifests as decreased cartilage cell apoptosis (Fig. 5A), decreased ROS release (Fig. 5B), increased cartilage cell number (Fig. 5C), significantly increased molecular expression beneficial to cartilage cells, significantly decreased molecular expression that damages cartilage cells (Fig. 5D), and restored chondrocyte proliferation ability (Fig. 5E). Notably, after inhibiting NF-κB function in TREM2-KO macrophages, CXCL3 expression was significantly downregulated (Fig. 5D). This indicates that TREM2 regulates NF-κB activity to influence CXCL3 expression, thereby altering the function of chondrocytes (Fig. 5D-F). We then use small interfering RNA (siRNA) to lower the expression level of CXCL3 in macrophages and evaluate its impact on chondrocyte function. The result revealed that it produced the same effect as mentioned earlier using the NF-κB inhibitor. This manifests as reduced chondrocyte apoptosis (Fig. 5G), decreased ROS release (Fig. 5H), and restored chondrocyte proliferation capacity (Fig. 5I). The above results indicate that TREM2 regulates the function of synovial macrophages on chondrocytes in OA by modulating NF-κB/CXCL3.

### Increasing TREM2 level is beneficial for improving OA treatment

**Figure 6:**
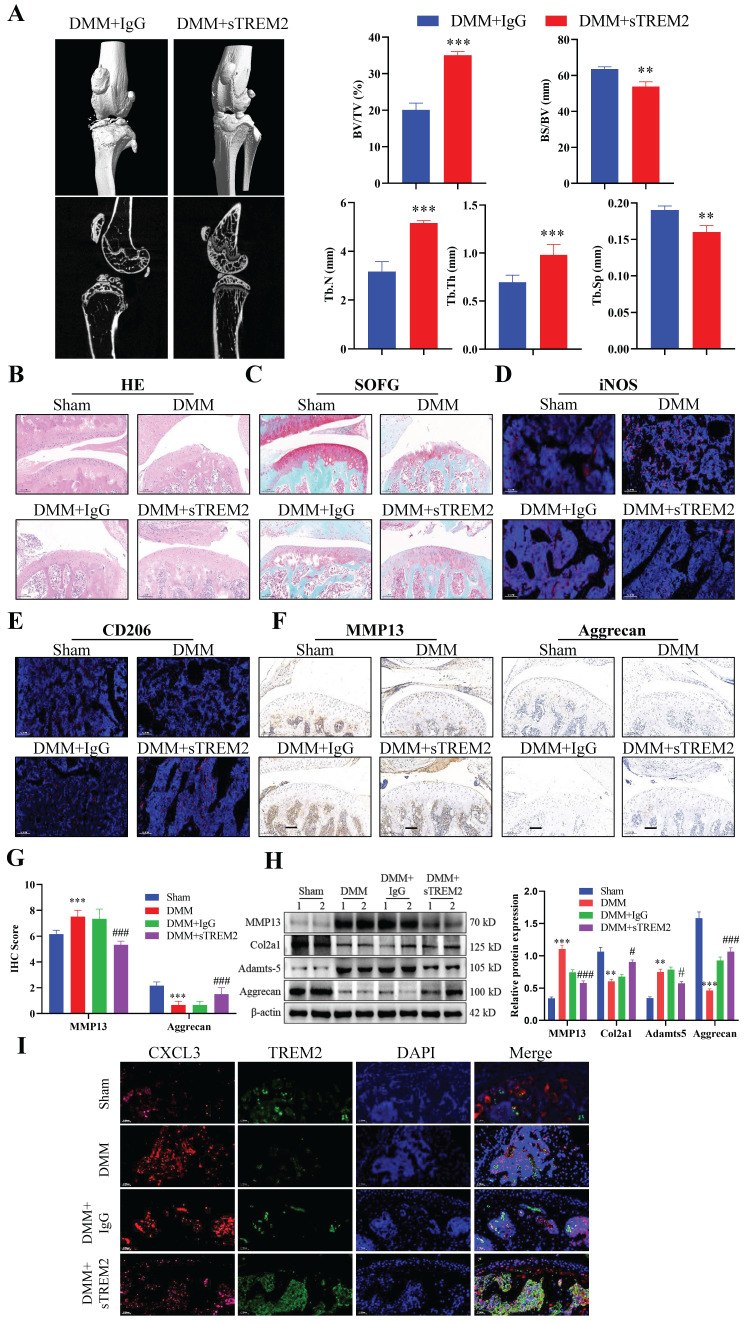
** Increasing the TREM2 level is beneficial for improving OA treatment.** A, Construct DMM mouse models, tail vein injection sTREM2 recombinant protein. Representative images of the microstructure of mouse bones were obtained in each group using Micro-CT. The right-hand side represents the statistical charts of the testing results for bone tissue and trabecular bone, including BV/TV, BS/BV, Tb-N, Tb-Th, and Tb-Sp. **P < 0.01, ***P < 0.001, compared to the DMM + IgG group. B-C, Representative images of SOFG and H&E staining for each group of mice. Scale bar: 100 μm. D-E, The representative immunofluorescence images of iNOS and CD206 positive cells in the synovium of the four groups of mice. Scale: 100 μm. F-G, Representative images and quantitative statistical results of MMP13 immunohistochemical staining in each group of mice. ***P < 0.001, compared to the Sham group, ^###^*P* < 0.001, compared to the DMM + IgG group. Scale: 100 μm. H, Western blotting is used to detect the protein content related to the extracellular matrix function in various groups of mouse chondrocytes. The graph on the right indicates the relative protein content statistics. **P < 0.01, ***P < 0.001, compared to the Sham group, ^#^*P* < 0.05, ^###^*P* < 0.001, compared to the DMM + IgG group. I, Representative immunofluorescence images of CXCL3 and TREM2 in synovial macrophages from each group of mice, as well as representative immunofluorescence images showing their co-localization on macrophages. DAPI staining indicates the cell nucleus. Scale: 100 μm.

After developing the OA mouse model, we injected sTREM2 recombinant protein through the tail vein injection to increase the TREM2 level in their bodies to further investigate the therapeutic effect of TREM2 on OA mice. Micro-CT results revealed that mice with sTREM2 supplementation exhibit significant improvement in OA compared to the control group (Fig. 6A). Conversely, sTREM2 increases bone formation and decreases bone resorption, thereby promoting an increase in bone mass, manifested as increased BV/TV, decreased BS/BV, increased Tb-N and Tb-Th, and decreased Tb-Sp (Fig. 6A). As compared with the isotype control group (IgG group), and the sTREM2 group exhibits significantly reduced synovial hyperplasia and cellular infiltration (Fig. 6B--C). Meanwhile, M1 macrophages decreased and M2 macrophage increased (Fig. 6D--E). Additionally, we revealed that sTREM2 supplementation significantly reduced the content of MMP13 in the organization closely related to OA development and increased the content of Aggrecan (Fig. 6F--G). Concurrently, the western blot results also obtained similar evidence (Fig. 6H, S2D). Moreover, the increased TREM2 level significantly promoted anti-inflammatory factors expression, while inhibiting pro-inflammatory factors expression, simultaneously enhancing Arg-1 expression and inhibiting iNOS and CXCL3 expressions (Fig. S2E). Additionally, the immunofluorescence results revealed that sTREM2 supplementation significantly reduced the content of CXCL3 (Fig. 6I). The above results indicate that supplementing sTREM2 significantly alleviate joint injury in mice with OA, promote synovial macrophage polarization from M1 to M2, suppress OA inflammatory response, and improve OA treatment.

## Discussion

This study has revealed the important role of TREM2 in OA pathogenesis and progression. Firstly, we conducted preliminary research on the role of TREM2 in OA using WT and TREM2-KO mice. The results showed that TREM2 expression was significantly downregulated in OA mice, and there was an increase in M1 macrophages and a decrease in M2 macrophages. TREM2-KO mice, however, aggravated joint damage and inflammation in OA mice, further reducing M2 macrophages and increasing M1 macrophages. Afterwards, we used GO analysis and KEGG pathway enrichment analysis to screen the potential mechanism of TREM2's influence on OA. Subsequently, we explored its mechanism using an NF-κB inhibitor and si-CXCL3 plasmid. Finally, we directly injected recombinant sTREM2 protein into OA mice through the tail vein injection method to evaluate the role of TREM2 in the OA mouse model. The results showed that the mice in DMM group and IgG group worsened in OA symptoms, while the mice in sTREM2 group showed an improvement in OA symptoms. Moreover, TREM2 inhibits CXCL3 expression by suppressing the PI3K/AKT and NF-κB signal transduction in synovial tissue and reprograms macrophage polarization by transforming M1 cells into M2 cells, thereby inhibiting inflammatory reactions and improving OA. Therefore, the reprogramming of polarization from the M1 to M2 macrophage by TREM2 is a potential therapeutic target for OA.

Maintaining cartilage function plays an important role in improving OA, zhang et al. found that the degradation of HIF-1α plays a key role in the cartilage degeneration of OA mice, proving that the stability of HIF-1α can delay the progression of OA^39^. In recent years, there is increasing evidence indicating that the imbalance of macrophage M1/M2 polarization plays a crucial role in maintaining the chondrocyte function in OA^7^, ^40^-^43^. A previous study revealed that M1 polarized macrophages, rather than M2, accumulated in the synovial tissues of humans and mice with OA^12^. M1 macrophage polarization enhances the release of inflammation factors, such as IL-6, IL-1β, MMPs, and TNF-α, thereby exacerbating the inflammatory response. Concurrently, M1 macrophages affect cartilage degradation and bone spur formation, thereby aggravating OA progression^12^, ^44^. On the contrary, M2 macrophage polarization increases the release of anti-inflammatory factors (such as IL-10 and IL-4), thereby improving the pathological state of OA^7^, ^42^. Therefore, inhibiting the macrophage differentiation into the M1 type and promoting their differentiation into the M2 type alleviate pathological changes and slow down OA progression, thereby potentially becoming an effective treatment strategy for OA.

TREM2 belongs to the immunoglobulin superfamily and is a transmembrane receptor^22^. Previous studies on macrophages have revealed that TREM2 binds to the adaptor proteins, DNAX activation protein 12 (DAP12) and DAP10, through residues of opposite charge in its transmembrane domain^45^. Upon ligand binding, the co-receptor molecule is phosphorylated, thereby recruiting intracellular signaling molecules. DAP12, also known as TYRO protein tyrosine kinase binding protein, mediates spleen tyrosine kinase activation, while DAP10 promotes signaling transduction by recruiting PI3K^46^, ^47^. TREM2 participates in clearing apoptotic cells and shows an anti-inflammatory effect, such as microglia and macrophages that promote anti-inflammatory gene expression in a TREM2-dependent manner^48^, ^49^. TREM2 signaling promotes macrophage survival when growth factors are depleted in the extracellular environment, and it can also improve macrophage survival in stressful environments such as tissue damage and inflammation *in vivo*^50^. This study indicates that TREM2 alleviates cartilage injury and synovitis in OA mice. Moreover, TREM2 converts M1 polarized macrophages into M2 macrophages and promotes anti-inflammatory cytokine expression during the OA process.

The newly emerged related research emphasizes the important roles of p38/ERK MAPK and p65/NF-κB signaling in macrophage activation and cartilage degradation, which promote OA pathogenesis and progression^51^, ^52^. A recent study has revealed that ERK, p38, and JNK signaling pathway activation enhances IL-6 and TNF-α production in human synovial fibroblasts^53^. Kim et al. confirmed that inhibiting the p38 MAPK signaling pathway hindered apoptosis in human OA chondrocytes^54^. Chen et al. revealed that blocking NF-κB suppressed IL-1β-induced inflammatory factor expression in OA chondrocytes and showed a protective effect on a mouse model of OA^55^. Moreover, NF-κB and MAPK activation are involved in LPS-induced iNOS expression and macrophage polarization^33^. In this study, we identified the significant role of NF-κB signaling in macrophage activation and chondrocyte proliferation and apoptosis. Consistent with previous studies, we revealed NF-κB signaling activation in LPS and IFN-γ-induced M1 polarization of macrophages and high expression during OA development^40^, ^56^. After inhibiting the NF-κB signaling function of macrophages with BAY 11-7082, we observed significant relief of OA, which was manifested as Col2a1 and Sox9 expression upregulation that protect cartilage, and Cox-2, MMP13, and Adamts-5 expression downregulation that have destructive functions. Additionally, chondrocyte apoptosis was decreased, while its proliferation was significantly increased. Concurrently, knocking down CXCL3 in macrophages produced similar effects. The above results further confirm that TREM2 promotes M2 macrophage polarization and maintains chondrocyte proliferation, inhibits apoptosis, alleviates inflammation reaction, and thus improves the pathological status of OA by regulating the NF-κB/CXCL3 axis.

Currently, the global population is entering an aging stage. The number and proportion of elderly populations in almost every country in the world are increasing. At the same time, the risk of elderly populations suffering from bone injuries is greater, which has become an important factor affecting their health and quality of life. OA leads to damage to the cartilage and surrounding tissues, causing adverse effects such as pain, stiffness, and loss of joint function for patients. Joint replacement surgery is currently the only effective treatment for severe joint damage caused by late-stage OA^57^, but it also has many drawbacks, such as postoperative pain ranging from moderate to severe, severely affecting patient recovery, patient satisfaction, and overall therapeutic effectiveness. In order to reduce the various risks brought by joint replacement, multiple biomaterials have been developed to promote bone tissue repair^58^. With the advancement of bone tissue engineering, the focus of research has shifted towards the development of bioactive biomaterials 59_._ However, many biomaterials have not yet achieved satisfactory therapeutic effects. In recent years, advancements in bone immunology research have revealed that the immune system, with macrophages as a key component, plays a crucial regulatory role in bone regeneration 60. This study explores how TREM2 induces macrophage polarization from M1 to M2, improves chondrocyte function in OA, reduces inflammation, and improves OA. Furthermore, the application of sTREM2 in OA mice demonstrates specific therapeutic effects, thereby establishing that TREM2 is likely an crucial target for regulating the continued progression of early-stage OA.

## Conclusion

**Figure 7:**
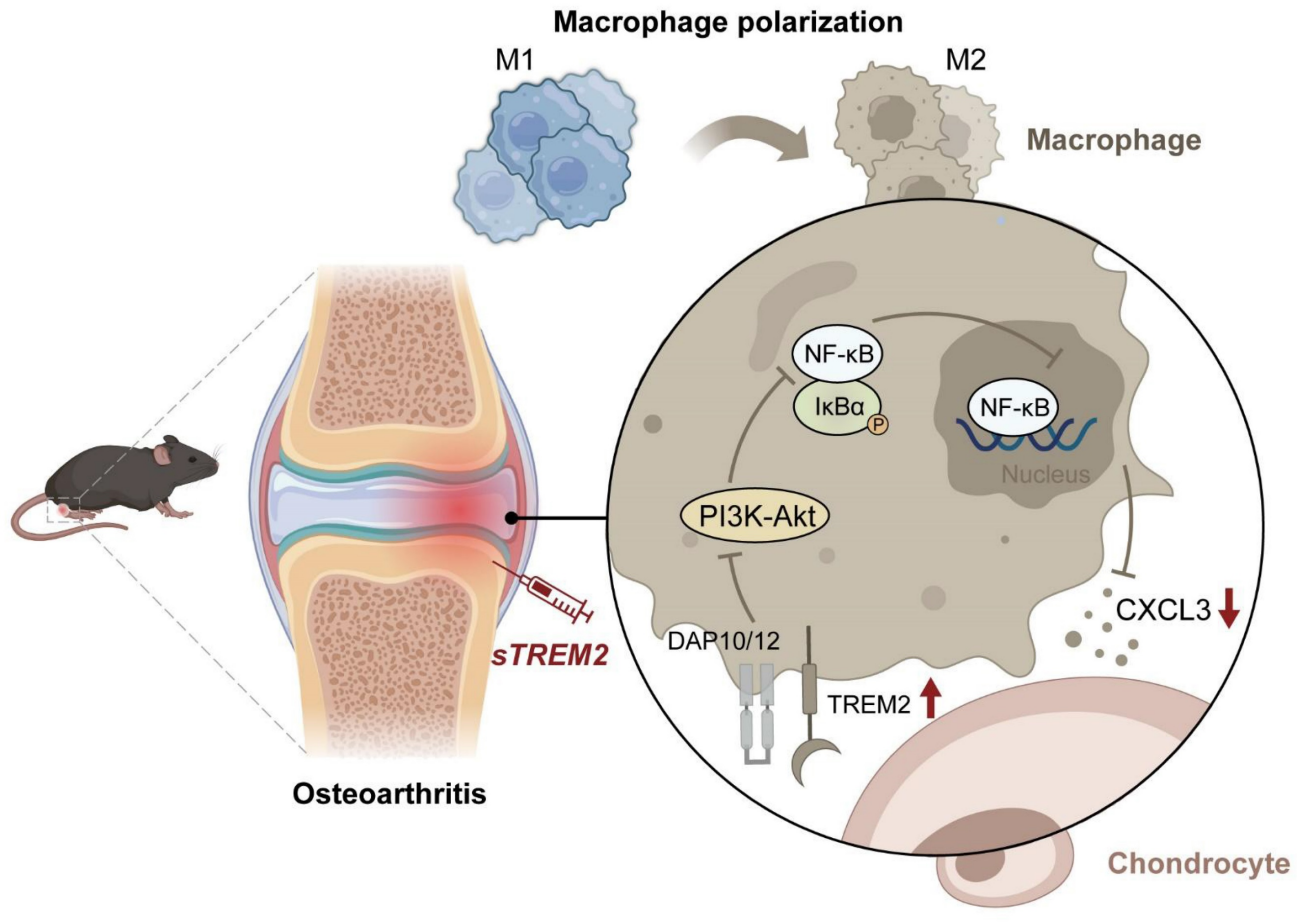
** The mechanism of TREM2 increases macrophage polarization from M1 to M2 and decreases OA.** Reduced TREM2 expression occurs during OA development, thereby increasing M1 macrophages. Supplementing sTREM2 can transition the polarization phenotype of macrophages from the M1 to the M2 subtype, thereby inhibiting PI3K/AKT and NF-κB signaling transduction in macrophages within the synovial tissue. This downregulates NF-κB and decreases CXCL3 expression, thereby preventing cartilage degeneration, suppressing inflammation response, and improving OA.

In summary, this study has revealed the crucial role of TREM2 in OA pathogenesis and progression. TREM2 switches the polarization phenotype of macrophages from M1 to M2 subtype and downregulates NF-κB signaling to reduce CXCL3 expression, thereby preventing cartilage degeneration and inhibiting inflammatory response. This indicates targeting macrophage transformation reprogramming through TREM2 as an effective preventive strategy for OA development (Fig. 7).

## Supplementary Material

Supplementary figures.
