# Supplementary material for: TREM2 promotes macrophage polarization from M1 to M2 and suppresses osteoarthritis through the NF-κB/CXCL3 axis
Source: Int J Biol Sci. 2024 Mar 11;20(6):1992–2007. doi: 10.7150/ijbs.91519 (PMC11008261; doi:10.7150/ijbs.91519)

Supplementary information

Supplementary Figure 1. The OA mouse model was developed using the DMM method. A, The representative images of H&E staining of the Sham and DMM groups of mouse synovial membrane tissue. B, The representative images of SOFG staining of Sham and DMM mouse cartilage tissues. C, The Sham and DMM groups were evaluated using the ICRS scoring system in mice. D, Western blotting is used to detect the expression level of Col1a1 in the synovial tissue of mice from the Sham and DMM groups. The endogenous control used was β-actin. 1 and 2 represent the different samples taken. The right figure quantitatively shows the relative expression levels of different proteins related to β-actin in the samples. ***$P < 0.001$ compared to the Sham 1 group; $^{\#\#\#}P < 0.001$ compared to the Sham 2 group.

Supplementary Figure 2. Cartilage function related protein detection. A, Western blotting detects the protein expression levels of matrix metalloproteinase 13 (MMP13), which promotes matrix degradation, and the marker for chondrocyte hypertrophy and calcification, collagen X (ColX), in the synovial tissue of the four groups of mice. The endogenous control used was β-actin. ***$P < 0.001$ compared to the WT + Sham group, $^{\#\#\#}P < 0.001$ compared to the TREM2-KO + Sham group. B, Immunohistochemical images of MMP13 and Aggrecan in the synovial tissue of the four groups of mice. Scale: 100 μm. C, Relative quantitative immunohistochemical analysis results. ***$P < 0.001$, compared to the WT + Sham group, $^{\#\#\#}P < 0.001$, compared to the TREM2-KO + Sham group. D, Construct DMM mouse models, tail

vein injection sTREM2 recombinant protein. Western blotting detects the protein content of Col1a1 in the synovial tissue of each group of mice. The endogenous control used was β-actin. 1 and 2 represent the different samples. E, Western blotting was used to detect the expression levels of inflammatory cytokines and macrophage polarization markers (iNOS and Arg-1) in the synovial tissue of the four groups of mice.

Supplementary Figure. 1

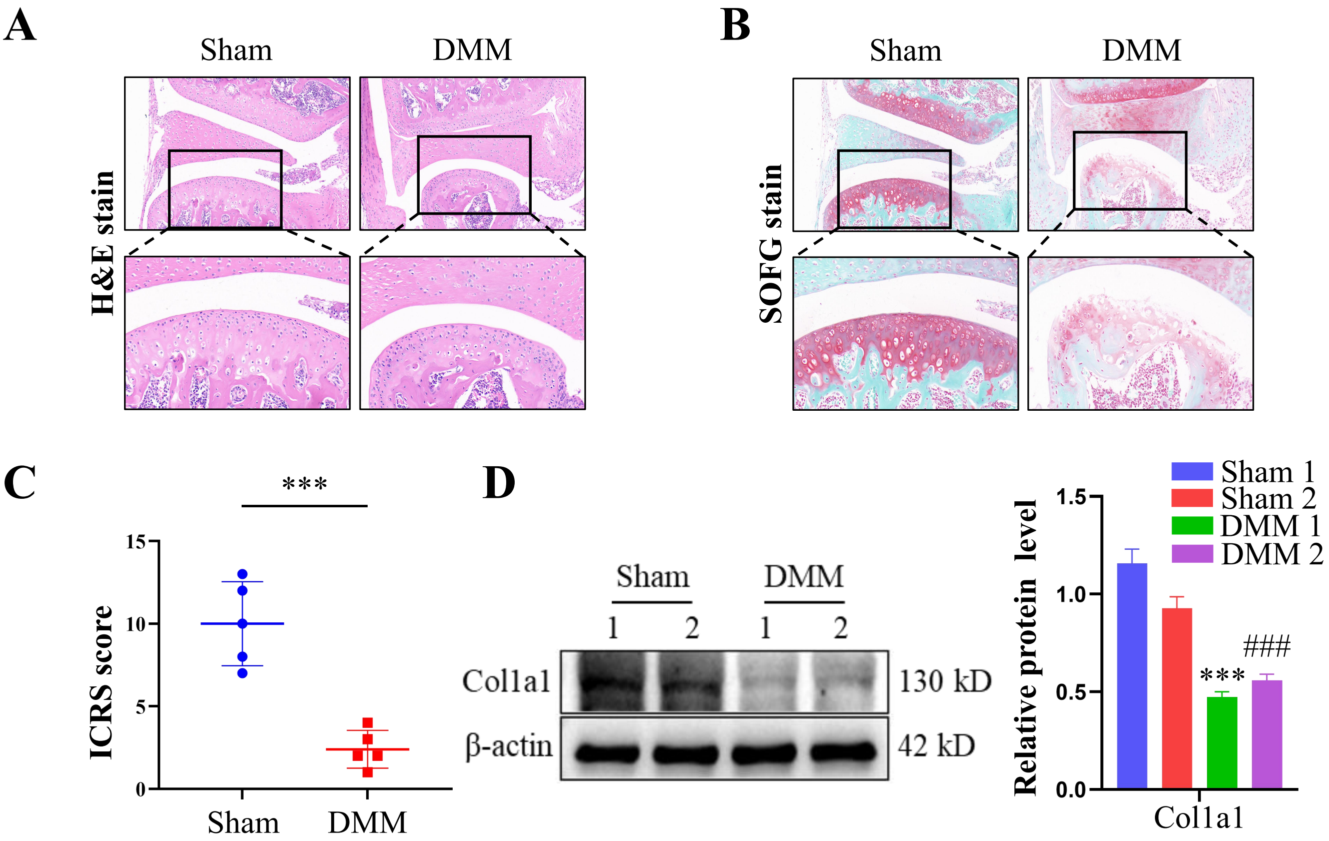

Supplementary Figure. 2

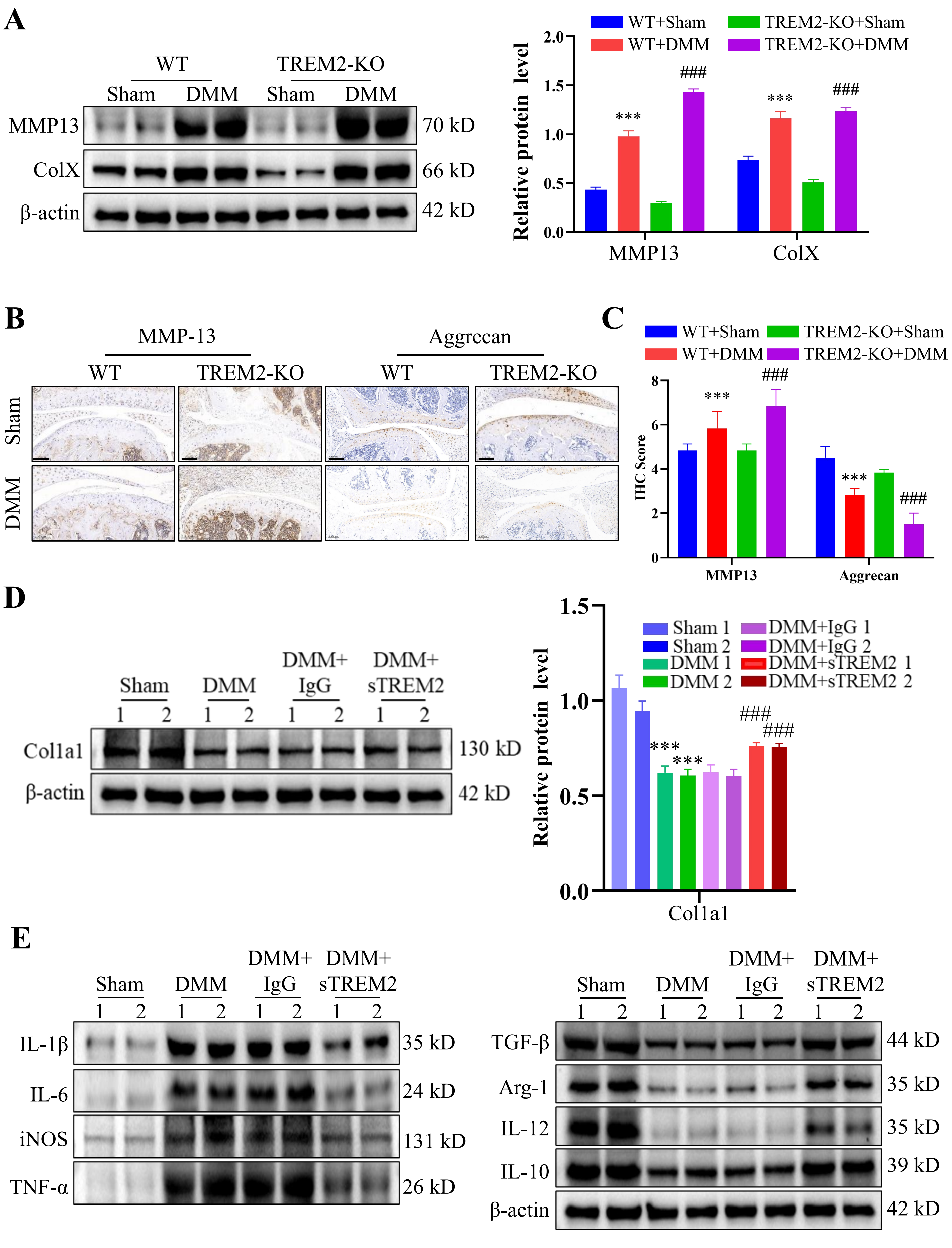

Supplement: Supplementary file 1 — Supplementary figures. [file ijbsv20p1992s1.pdf]
